# Periprocedural Direct Oral Anticoagulant Management: The RA-ACOD Prospective, Multicenter Real-World Registry

**DOI:** 10.1055/s-0040-1712476

**Published:** 2020-06-26

**Authors:** Raquel Ferrandis, Juan V. Llau, Javier F. Sanz, Concepción M. Cassinello, Óscar González-Larrocha, Salomé M. Matoses, Vanessa Suárez, Patricia Guilabert, Luís-Miguel Torres, Esperanza Fernández-Bañuls, Consuelo García-Cebrián, Pilar Sierra, Marta Barquero, Nuria Montón, Cristina Martínez-Escribano, Manuel Llácer, Aurelio Gómez-Luque, Julia Martín, Francisco Hidalgo, Gabriel Yanes, Rubén Rodríguez, Beatriz Castaño, Elena Duro, Blanca Tapia, Antoni Pérez, Ángeles M. Villanueva, Juan-Carlos Álvarez, Sergi Sabaté, for the RA-ACOD investigators

**Affiliations:** 1 Anaesthesiology and Critical Care, Hospital Universitari i Politècnic La Fe, València, Spain; 2 Anaesthesiology and Critical Care, Hospital Universitario Doctor Peset, València, Spain; 3 Anaesthesiology and Critical Care, Hospital General Universitario Gregorio Marañón, Madrid, Spain; 4 Anaesthesiology and Critical Care, Hospital Miguel Servet, Zaragoza, Spain; 5 Anaesthesiology and Critical Care, Hospital Galdakao-Usánsolo, Bizkaia, Spain; 6 Anaesthesiology and Critical Care, Hospital Universitario de Gran Canaria Doctor Negrín, Las Palmas de Gran Canaria, Spain; 7 Anaesthesiology and Critical Care, Hospital Universitari Vall d'Hebron, Barcelona, Spain; 8 Anaesthesiology and Critical Care, Hospital Universitario Puerta del Mar, Cadiz, Spain; 9 Anaesthesiology and Critical Care, Hospital Lluís Alcanyís, Xàtiva, Spain; 10 Anaesthesiology and Critical Care, Fundació Puigvert, Barcelona, Spain; 11 Anaesthesiology and Critical Care, Hospital Parc Taulí, Sabadell, Spain; 12 Anaesthesiology and Critical Care, Hospital de La Ribera, Alzira, Spain; 13 Anaesthesiology and Critical Care, Hospital Costa del Sol, Marbella, Spain; 14 Anaesthesiology and Critical Care, Hospital Universitario Virgen de la Victoria, Málaga, Spain; 15 Anaesthesiology and Critical Care, Clínica Universitaria de Navarra, Pamplona, Spain; 16 Anaesthesiology and Critical Care, Hospital Virgen del Rocio, Sevilla, Spain; 17 Anaesthesiology and Critical Care, Hospital Universitario de Móstoles, Madrid, Spain; 18 Anaesthesiology and Critical Care, Complejo Hospitalario de Toledo, Toledo, Spain; 19 Anaesthesiology and Critical Care, Hospital Universitario de Getafe, Madrid, Spain; 20 Anaesthesiology and Critical Care, Hospital La Paz, Madrid, Spain; 21 Anaesthesiology and Critical Care, Hospital de Mataró, Mataró, Spain; 22 Anaesthesiology and Critical Care, Hospital Universitario Marqués de Valdecilla, Santander, Spain; 23 Anaesthesiology and Critical Care, Hospital Universitario Parc de Salut Mar, Barcelona, Spain

**Keywords:** anticoagulant, dabigatran etexilate, apixaban, rivaroxaban, perioperative period

## Abstract

**Introduction** There is scarce real-world experience regarding direct oral anticoagulants (DOACs) perioperative management. No study before has linked bridging therapy or DOAC-free time (pre-plus postoperative time without DOAC) with outcome. The aim of this study was to investigate real-world management and outcomes.

**Methods** RA-ACOD is a prospective, observational, multicenter registry of adult patients on DOAC treatment requiring surgery. Primary outcomes were thrombotic and hemorrhagic complications. Follow-up was immediate postoperative (24–48 hours) and 30 days. Statistics were performed using a univariate and multivariate analysis. Data are presented as odds ratios (ORs [95% confidence interval]).

**Results** From 26 Spanish hospitals, 901 patients were analyzed (53.5% major surgeries): 322 on apixaban, 304 on rivaroxaban, 267 on dabigatran, 8 on edoxaban. Fourteen (1.6%) patients suffered a thrombotic event, related to preoperative DOAC withdrawal (OR: 1.57 [1.03–2.4]) and DOAC-free time longer than 6 days (OR: 5.42 [1.18–26]). Minor bleeding events were described in 76 (8.4%) patients, with higher incidence for dabigatran (12.7%) versus other DOACs (6.6%). Major bleeding events occurred in 17 (1.9%) patients. Bridging therapy was used in 315 (35%) patients. It was associated with minor (OR: 2.57 [1.3–5.07]) and major (OR: 4.2 [1.4–12.3]) bleeding events, without decreasing thrombotic events.

**Conclusion** This study offers real-world data on perioperative DOAC management and outcomes in a large prospective sample size to date with a high percentage of major surgery. Short-term preprocedural DOAC interruption depending on the drug, hemorrhagic risk, and renal function, without bridging therapy and a reduced DOAC-free time, seems the safest practice.

## Introduction

Direct oral anticoagulants (DOACs) are a group of agents acting against specific coagulation targets IIa (dabigatran etexilate) and Xa (apixaban, edoxaban, and rivaroxaban). 1 2 The perioperative management of anticoagulant drugs is always challenging. For decades, the protocol for patients on vitamin K antagonists (VKA) has included a bridging therapy, mostly a low-molecular weight heparin (LMWH), but current guidelines recommend against bridging in low to moderate thrombotic-risk patients. 3 4 5

During the first years of DOAC management, due to the lack of experience and unsuitable standardized monitoring, dissimilar recommendations were made, and some of them (such as the Spanish ones) proposed bridging therapy for selected patients at high thrombotic risk. 6 7 8 However, pharmacokinetic characteristics of DOACs seem to favor discontinuing the drug for some days (depending on the DOAC, the hemorrhagic risk, and renal function) without bridging, which has been included in the latest proposals. 5 9 10

In a recent meta-analysis, 11 including the Phase III studies of each drug, 12 13 14 15 the rate of 30-day postoperative arterial thromboembolic events (all causing ischemic stroke) was 0.41 and 1.81% for major bleeding events, but the meta-analysis does not differentiate the outcome in relation to perioperative DOAC withdrawal. A previous large real-world study, 16 based on 863 procedures, reported major cardiovascular events at 1.0%, up to 4.6% when considering only the 10.1% major surgeries. Similarly, major bleeding events were more common after major procedures (8.0% compared with 1.2% in the pooled data). The PAUSE study, 17 using a standardized perioperative DOAC management, based on DOAC pharmacokinetic properties, procedure-associated bleeding risk, and renal function, without bridging therapy, showed low rate of major bleeding and arterial thrombotic events. But none of these studies has linked complications in periprocedural DOAC management with drug-free time or bridging use.

In order to know real-world management and outcomes, we conducted an open, prospective, observational nonintervention registry.

## Methods

The RA-ACOD is a prospective, observational, Spanish multicenter registry of adult patients under DOAC treatment requiring either scheduled or urgent surgery or an invasive procedure. No specific DOAC management protocol was standardized. The present study analyses perioperative DOAC management for scheduled procedures.

### Ethics Approval/License

The Clinic Hospital of València Ethics Committee first approved the RA-ACOD study on February 27, 2014 and it was registered at ClinicalTrials.gov (NCT03182218). Each hospital local committee also approved the study. All patients provided written informed consent, including a data protection waiver, before enrolment.

### Patient Population

The RA-ACOD registry was open to all the Anesthesiology Departments of Spanish hospitals. Consecutive patients were eligible if the inclusion criteria were met: age: 18 years or older; chronic treatment with any DOAC (at least 3 months); scheduled surgery or invasive procedure; signed and dated informed consent form; availability for follow-up by telephone call. No exclusion criteria were applied.

### Aim and Outcomes

The aim of the study was to evaluate current practice in scheduled perioperative DOACs management to elaborate future safety and efficacy recommendations based on the hemorrhagic and thrombotic events found.

The primary outcomes were the cumulative incidence of periprocedural thrombotic (arterial or venous thrombotic events) or hemorrhagic (major and minor bleeding events) complications. The secondary outcomes were the relationship between events incidence and the use of bridging therapy and to obtain information on current DOACs management in clinical practice.

### Data Reporting

Reported variables and definitions are summarized in Supplementary Material S1 and Table S1 . For the statistical analysis, two groups of thrombotic risk were made according to clinical relevance: low-moderate and high thrombotic risk. Taking into account the type of surgeries, patients were divided into two groups: low and moderate-high bleeding risk. In addition, we analyzed the interruption of DOAC based on estimated glomerular filtration rate (eGFR), and we divided all patients into two groups according to eGFR more or less and equal than 50 mL kg 1.73 m ^−2^ .

About the perioperative DOAC management both the time of DOAC withdrawal (preoperative) and the time to DOAC restart (postoperative) have been considered. Furthermore, taking these two times together, that is, the total time without DOAC, has been named as DOAC-free time.

Arterial and venous thrombotic events were diagnosed by physicians not related to the study. The anesthesiologist and the surgeon in charge assessed whether the intraoperative bleeding in the operating room was “more than normal.” Bleeding events were classified as *major* (any fatal hemorrhage, or related to a decrease in hemoglobin >2 g/dL, or requiring reintervention) or *minor* (any hemorrhagic event not classified as major). 18 The cause of death was recorded and its association with hemorrhagic or thrombotic etiology when applicable.

There were two follow-up frames: the immediate postoperative period (24–48 hours) and up to 30 days postoperatively. For the 30-day assessment, a double system was established: telephone interview and clinical history review. The information was obtained by a member of the local research team. To prevent variability in data collection, a centralized database with quality-control algorithms was developed. All the investigators were trained to fill in the electronic case report form and to collect data to uniform criteria, especially regarding definitions of the medical conditions.

### Statistics

Categorical variables are expressed as absolute and relative frequencies, with 95% confidence intervals (CIs) for proportions based on the binomial distribution. Quantitative continuous variables (age, weight, and height) are shown as mean (standard deviations). All other quantitative variables, due to their measure scale and strong asymmetry, are studied using nonparametric techniques: median (interquartile range).

Chi-square test was performed to find possible associations of those variables with the main outcome parameters (thrombosis, bleeding, and mortality). Bivariate odds ratios (ORs) and 95% CIs were also calculated. Kruskal-Wallis and U Mann-Whitney test were used to find possible associations with the main outcomes. Adjusted *p* -values are obtained using Holm multiple comparison procedure.

Logistic regression with stepwise (forward/backward) covariate selection was used to study the possible relationship between the outcome parameters (thrombotic event, minor and major bleeding event, and mortality) and DOAC type, thrombotic risk, hemorrhagic risk, withdrawal time, DOAC-free time, and bridging therapy. Independent covariates were entered into the model if a significant association was found ( *p* < 0.05). Covariates were sequentially removed if this exclusion did not result in a significant change in the log-likelihood ratio test. The cutoff for variable removal was set at a significance level of 0.1. We then calculated the adjusted ORs and the corresponding 95% CI values. The calibration of the logistic regression model was assessed by the Hosmer–Lemeshow goodness-of-fit statistic.

A result was considered statistically significant when the *p* -value (or adjusted *p* -value, in case of multiple comparisons) was less than 0.05. The statistical analysis was performed with the statistical package R (version 3.2.2; R: A language and environment for statistical computing, https://www.r-project.org/ ).

## Results

### Cohort Characteristics

**Fig. 1:**
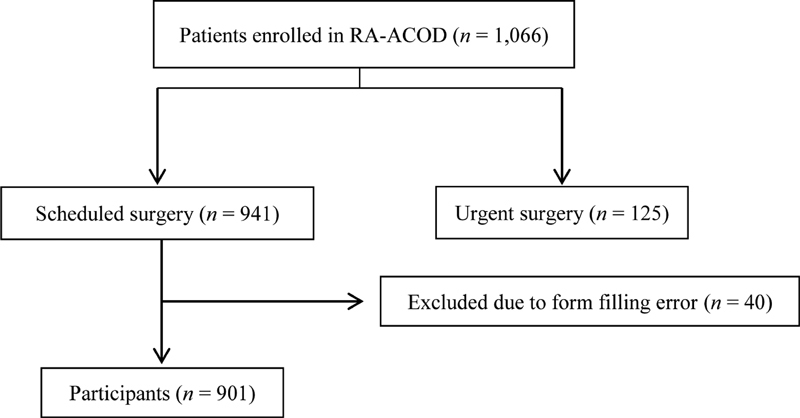
Recruitment flowchart.

RA-ACOD registry enrolled 1,066 patients in 26 Spanish hospitals ( Supplementary Table S2 ) from November 2014 till December 2017. Nine hundred forty-one of them who underwent scheduled surgery, were included in the present study, 40 were excluded because of a form filling error. Thus, we analyzed data obtained from 901 patients ( Fig. 1 ). Among the patients included, 322 (35.7%) were on apixaban, 304 (33.7%) on rivaroxaban, 267 (29.6%) on dabigatran, and 8 (0.9%) on edoxaban. The clinical characteristics of these patients are given in Table 1 and Supplementary Table S3 . Types of surgery included in the study are listed in Supplementary Table S4 .

**Table 1:** Patients and procedures characteristics

Total subjects ( *n* )		901
Demographic data	Gender, male [ *n* (%)]	553 (61.4)
	Age [y, median (25–75%)]	75 (69–81)
	Weight [kg, median (25–75%)]	79 (70–89)
	Height [cm, median (25–75%)]	165 (159–172)
	eGFR [median (25–75%)]	77.9 (58.4–101.3)
Comorbidity [ *n* (%)]	History of heart failure	81 (9.0)
	Hypertension	731 (81.1)
	Diabetes mellitus	284 (31.5)
	Stroke	200 (22.2)
	Pulmonary embolism	14 (1.6)
	Deep vein thrombosis	33 (3.7)
	Peripheral artery disease	178 (19.8)
	Active oncologic disease	173 (19.2)
	Thrombophilia	8 (0.9)
	Liver disease	36 (4.0)
	Alcohol abuse	73 (8.2)
	Street drug addiction	3 (0.3)
DOAC indication [ *n* (%)]	Subjects (n)	901
	Atrial fibrillation	874 (97.0)
	Venous thromboembolism	15 (1.7)
	Recurrent venous thromboembolism	12 (1.3)
Concomitant treatment [ *n* (%)]	Aspirin	77 (8.5)
	Clopidogrel	11 (1.2)
	NSAID	13 (1.4)
	Amiodarone	80 (8.9)
	Verapamil	7 (0.8)
	Ketoconazole	1 (0.1)
	Dronedarone	7 (0.8)
	Other *p* -glycoprotein inhibitors	54 (6.0)
CHA _2_ DS _2_ -VASc	Subjects ( *n* ) ^a^	874
	0–4	613 (70.1)
	5–6	212 (24.3)
	7–9	49 (5.6)
Patient thrombotic risk ^b^ [ *n* (%)]	Subjects ( *n* )	901
	Low	619 (68.7)
	Moderate	227 (25.2)
	High	55 (6.1)
Bleeding risk of procedure ^b^ [ *n* (%)]	Subjects ( *n* )	901
	Low	412 (45.7)
	Moderate	427 (47.4)
	High	62 (6.9)
Type of surgery [ *n* (%)]	Subjects ( *n* )	901
	Minor	235 (26.1)
	Major nonorthopaedic	304 (33.7)
	Major orthopaedic	178 (19.8)
	Endoscopy	51 (5.7)
	Ophthalmologic	96 (10.7)
	Others	37 (4.1)
Type of anaesthesia [ *n* (%)]	Subjects ( *n* )	901
	General	515 (57.0)
	Neuraxial block	229 (25.5)
	Nerve block	72 (8.0)
	Local	85 (9.5)

Abbreviations: CHA _2_ DS _2_ -VASc score,1 point for congestive heart failure, hypertension, diabetes mellitus, female, age 65–74 y and vascular disease (peripheral artery disease, myocardial infarction, aortic plaque); 2 points for age ≥75 y, prior stroke or transient ischemic attack or thromboembolism; DOAC, direct oral anticoagulant; eGFR, estimated glomerular filtration rate; NSAID, nonsteroidal anti-inflammatory drug.

a Only patients with atrial fibrillation.

b See Supplementary Material S1 .

### Perioperative DOAC Management

Table 2 summarizes perioperative DOAC management, considering the type of DOAC and the use of bridging therapy. The (median [25th–75th percentile]) time of preoperative DOAC withdrawal was 2 days (2–3 days). The dabigatran withdrawal time was significantly longer by 1 day ( *p* = 0.013).

**Table 2:** Perioperative DOAC management

	All DOAC *n* = 901 ( *n* [%])	Dabigatran *n* = 267 ( *n* [%])	Apixaban *n* = 322 ( *n* [%])	Rivaroxaban *n* = 304 ( *n* [%])	Edoxaban *n* = 8 ( *n* [%])
Preoperative DOAC management					
Withdrawal time					
1 d	121 (13.4)	33 (12.4)	43 (13.4)	44 (14.5)	1 (12.5)
2 d	367 (40.7)	94 (35.2)	126 (39.1)	141 (46.4)	6 (75.0)
3 d	208 (23.1)	61 (22.8)	87 (27.0)	60 (19.7)	0
4 d	59 (6.5)	28 (10.5)	16 (5.0)	15 (4.9)	0
5 d	109 (12.1)	40 (15.0)	34 (10.6)	34 (11.2)	1 (12.5)
> 5 d	37 (4.1)	11 (4.1)	16 (5.0)	10 (3.3)	0
Bridging therapy					
No	586 (65.0)	167 (62.8)	219 (68.0)	191 (62.8)	8 (100.0)
Yes	315 (35.0)	99 (37.2)	103 (32.0)	113 (37.2)	0
LMWH prophylactic dose	261 (82.9)	78 (78.8)	91 (88.3)	92 (81.4)	0
LMWH therapeutic dose	54 (17.1)	21 (21.2)	12 (11.7)	21 (18.6)	0
Start 24 h after ACOD stop	286 (91.1)	89 (89.9)	91 (89.2)	106 (93.8)	0
Start 48 h after ACOD stop	28 (8.9)	10 (10.1)	11 (10.8)	7 (6.2)	0
Last dose 12 h before surgery	199 (63.8)	65 (66.3)	68 (67.3)	66 (58.4)	0
Last dose 24 h before surgery	113 (36.2)	33 (33.7)	33 (32.7)	47 (41.6)	0
Postoperative DOAC management					
Postoperative restart time					
< 24 h	210 (23.3)	60 (22.5)	95 (29.5)	53 (17.4)	2 (25.0)
= 24–48 h	295 (32.7)	85 (31.8)	96 (29.8)	109 (35.9)	5 (62.5)
= 3–30 d	319 (35.4)	107 (40.1)	94 (29.2)	118 (38.8)	0
No restart in 30 d	77 (8.5)	15 (5.6)	37 (11.5)	24 (7.9)	1 (12.5)
Bridging therapy					
No	403 (44.7)	107 (39.8)	167 (51.9)	123 (40.5)	6 (75.0)
Yes	498 (55.3)	160 (60.0)	155 (48.1)	181 (59.5)	2 (25.0)
LMWH prophylaxis dose	407 (45.2)	131 (49.2)	124 (38.5)	151 (49.7)	1 (12.5)
LMWH therapeutic dose	91 (10.1)	29 (10.9)	31 (9.6)	30 (9.9)	1 (12.5)
Perioperative DOAC management					
DOAC-free time (d) a	5 (3–8)	6 (4–9)	5 (3–7)	5 (3–8)	3.5 (3–4)

Abbreviations: DOAC, direct oral anticoagulant; DOAC-free time, time since preoperative withdrawal till postoperative restart; LMWH, low-molecular weight heparin.

a Median (25th–75th percentile).

We also analyzed the withdrawal time related to the eGFR and the bleeding risk of the surgery ( Table 3 ). No statistically significant differences were found among the four DOACs ( *p* = 0.767) when eGFR was ≥50 mL kg 1.73 m ^−2^ . Nevertheless, for an eGFR <50 mL kg 1.73 m ^−2^ , dabigatran was interrupted 1 day more than the other DOACs (4 days [3–5 days]).

**Table 3:** Preoperative DOAC-withdrawal time related to estimated glomerular filtration rate (eGFR) and bleeding risk of surgery

DOAC	Anti-Xa		Anti-II	
*n*	634		267	
eGFR (mL min 1.73 m ^−2^ )	≥50	<50	≥50	<50
*n*	528	106	244	23
Preop. bridging therapy [ *n* (%)]	176 (33.3)	40 (37.7)	85 (35.7)	12 (52.2)
Low bleeding risk of surgery	*n* = 247	*n* = 47	*n* = 110	*n* = 8
Withdrawal-time (d) ^a^	2 (1–3)	2 (2–3)	2.5 (1.3–4.5)
Moderate/high bleeding risk of surgery	*n* = 281	*n* = 59	*n* = 134	*n* = 15
Withdrawal-time (d) ^a^ rate (eGFR) and bleeding risk of surgery	2 (2–3)	3 (2–4)	4 (3–5)

Abbreviations: DOAC, direct oral anticoagulant; AntiXa, apixaban, rivaroxaban, edoxaban; Anti II, dabigatran; Preop, preoperative.

a Median (25th–75th percentile).

Preoperative bridging therapy with LMWH was used in 315 (35%) of the patients, 261 (82.7%) of them with prophylactic dose. Bridging therapy was more frequently used in patients who withdrew DOAC treatment more than 2 days (61% [252/413]), compared with patients who withdrew DOAC 2 days or less before surgery (12.9% [63/488]). However, bridging therapy indication was not related to the DOAC type ( *p* = 0.382), or thrombotic risk ( *p* = 0.103; 32.8% [203/619], 38.8% [88/227] and 43.6% [24/55] of low, moderate, and high-risk patients, respectively).

In the postoperative period, 56% (505/901) of the patients restarted DOAC in the first 48 hours and at least 75% of patients had done so within 5 days after surgery (2 days [1–5 days]). Postoperative thromboprophylaxis was used in 498 (55.3%) patients and, similar to the preoperative period, in most cases with prophylactic dose ( Table 2 ). Mechanical prophylaxis devices were used in 163 (18.1%) patients. Mechanical and pharmacological prophylaxis were used regardless of the time of DOAC restart.

During the 30-day follow-up, DOAC was not restarted in 77 (8.5%) patients. Postoperative thromboprophylaxis was prescribed in 68 of them; prophylactic LMWH in 46, and therapeutic in 22. There are several postoperative complications related to the delay in DOAC reinitiating ( Supplementary Material S2 for details).

### Periprocedural Events

The occurrences of thrombotic and bleeding events within the 30 days after surgery are shown in Table 4 ( Supplementary Tables S5 and S6 ). A thrombotic event was observed in 14 (1.6%, [0.9–2.6%]) patients: ischemic stroke in five (0.6%), acute coronary syndrome in six (0.7%), and venous thromboembolism (VTE) in three (0.3%) patients. Thrombotic events were not statistically related to the thrombotic risk classification. Also, the use of bridging therapy was not associated with a lower rate of thrombotic events. However, preoperative DOAC withdrawal time (OR: 1.57 [1.03–2.4]) and DOAC-free time longer than 6 days (OR: 1.9 [1.43–2.5]) were independent risk factors for thrombotic event. A preoperative DOAC withdrawal longer than 3 days and 4 days was statistically related to thrombotic event with an OR: 3.5 (1.2–10) and OR: 4 (1.3–11.7), respectively. The final model of the multivariate binary regression analysis showed that higher prevalence of thrombotic events was associated with a DOAC-free time longer than 6 days (OR: 5.42 [1.18–26]).

**Table 4:** Perioperative thrombotic and bleeding risk events (bivariate analysis)

	Total Patients	Thrombotic events			Minor bleeding events			Major bleeding events		
	*n*	*n* (%)	OR (95% CI)	*p* -Value	*n* (%)	OR (95% CI)	*p* -Value	*n* (%)	OR (95% CI)	*p* -Value
Bleeding risk Low Moderate/high	412 489	3 (0.7) 11 (2.3)	3.1 (0.9–11.3)	0.117	25 (6.1) 51 (10.4)	1.8 (1.1–3.0)	0.026	5 (1.2) 12 (2.5)	2.0 (0.7–5.9)	0.264
Thrombotic risk Low/moderate High	846 55	12 (1.4) 2 (3.6)	3.3 (0.4–16.7)	0.178	70 (8.3) 6 (10.9)	1.0 (0.4–2.5)	0.963	14 (1.7) 3 (5.5)	4.4 (0.9–18.6)	0.051
Preoperative withdrawal time (d)										
All DOAC 3 >3	696 205	7 (1) 7 (3.4)	3.5 (1.2–10)	0.033	48 (6.9) 28 (13.7)	2.1 (1.3–3.5)	0.004	12 (1.7) 5 (2.4)	1.42 (0.5–4)	0.136
Dabigatran 3 >3	188 79	2 (1.1) 3 (3.8)	3.7 (0.6–22.4)	0.313	18 (9.6) 16 (20.3)	2.4 (1.2–5.0)	0.029	1 (0.5) 2 (2.5)	4.9 (0.4–54.4)	0.436
Apixaban 3 >3	256 66	2 (0.8) 2 (3)	2.5 (0.9–6.8)	0.397	21 (8.2) 4 (6.1)	0.76 (0.3–1.9)	0.747	6 (2.3) 1 (1.5)	0.7 (0.1–4.3)	1
Rivaroxaban 3 >3	244 60	3(1.2) 2 (3.3)	2 (0.69–6.2)	0.555	9 (3.7) 8 (13.3)	2.6 (1.5–4.6)	0.009	5 (2) 2 (3.3)	1.5 (0.4–4.8)	0.9
Preoperative bridging therapy										
No Yes	586 315	7 (1.2) 7 (2.2)	1.9 (0.7–5.4)	0.366	37 (6.3) 39 (12.4)	2.1 (1.3–3.4)	0.003	5 (0.9) 12 (3.8)	4.6 (1.6–13.2)	0.004
LMWH prophylactic dose LMWH therapeutic dose	261 54	4 (1.5) 3 (5.6)	3.8 (0.8–17.4)	0.187	27 (10.3) 12 (22.2)	2.5 (1.2–5.3)	0.029	7 (2.7) 5 (9.3)	3.7 (1.1–12.1)	0.056
Postoperative restart DOAC (d)										
All DOAC 3 >3	561 340	5 (0.9) 9 (2.6)	3 (1.005–9)	0.07	32 (5.7) 44 (12.9)	2.4 (1.5–4)	0.000	5 (0.9) 12 (3.5)	4 (1.4–11.6)	0.01
Dabigatran 3 >3	158 107	2 (1.3) 1 (1.1)	2.3 (0.4–13.7)	0.658	12 (7.6) 22 (20.5)	3 (1.5–6.7)	0.004	1 (0.6) 2 (1.8)	3.5 (0.3–33)	0.733
Apixaban 3 >3	216 106	1 (0.5) 3 (2.8)	6.3 (0.6–61)	0.205	13 (6) 12 (11.3)	2 (0.87–4.5)	0.147	2 (0.9) 5 (4.7)	5.3 (1.01–27)	0.074
Rivaroxaban 3 >3	180 126	2 (1.1) 3 (2.3)	2.2 (0.3–13)	0.686	7 (3.9) 10 (7.9)	2 (0.8–5.7)	0.205	2 (1–1) 5 (3.9)	3.7 (0.7–19)	0.209
Postoperative thromboprophylaxis No Yes	403 498	3 (0.7) 11 (2.2)	3.0 (0.8–10.8)	0.136	15 (3.7) 61 (12.2)	3.6 (2.0–6.4)	0.000	5 (1.2) 12 (2.4)	2.0 (0.7–5.6)	0.303
LMWH prophylactic dose LMWH therapeutic dose	407 91	6 (1.5) 5 (5.5)	3.9 (1.2–13.0)	0.049	45 (11.1) 16 (17.6)	1.7 (0.9–3.2)	0.124	9 (2.2) 3 (3.3)	1.5 (0.4–5.7)	0.816
DOAC-free time (d)										
6 >6	525 376	3 (0.6) 11 (2.9)	1.9 (1.43–2.5)	0.006	26 (5) 50 (13.3)	1.66 (1.4–2)	0.000	6 (1.1) 11 (2.9)	1.56 (1.1–2.2)	0.08

Abbreviations: CI, confidence interval; DOAC, direct oral anticoagulant; LMWH, low-molecular weight; OR, odds ratio.

Intraoperative bleeding was assessed by the physician in charge in 899 patients and was considered higher than normal in 49 (5.5%) patients. This fact was well predicted by bleeding risk classification, showing an OR: 4.7 (2.2–10.2) in procedures of the moderate/high bleeding risk group compared with the low risk one. Patients with a higher bleeding assessment also had a statistically significant higher DOAC free time ( *p* < 0.001). Only five patients (0.6%) needed a surgical re-operation due to bleeding. The surgical procedures classified as moderate/high bleeding risk were associated with a statistically significant higher incidence of minor bleeding events (OR 1.8 [1.1–3.0]), but they were not associated with major bleeding events ( Table 4 ). Dabigatran showed a higher rate of minor bleeding events compared with the other type of DOAC (OR: 2.3 [1.3–4.1]). There was a statistically significant relationship between the use of preoperative bridging therapy and bleeding events, minor (OR: 2.1 [1.3–3.4]) and major (OR: 4.6 [1.6–13.2]). Likewise, there was an association between the longer preoperative withdrawal time and the incidence of minor bleeding events ( *p* < 0.001), finding a statistical interaction between DOAC withdrawal time and the use of bridging therapy (OR: 4.3 [2.2–8.7]). Among patients who suffered some minor bleeding event, the DOAC was restarted significantly later ( *p* < 0.001), especially in the case of dabigatran (3.1 days [1.5–6.4], *p* = 0.004). Applying the final model of the multivariate binary regression analysis the higher prevalence of major bleeding events was associated with bridging therapy (OR: 4.2 [1.4–12.3]) and the risk of high bleeding at surgery (OR: 5.3 [1.78–15.8]). If DOAC-free time longer than 6 days was added to the model, it was not significantly associated with major bleeding events.

In the 30-day follow-up, 14 (1.6%) patients died ( Supplementary Table S7 ). The death was considered of hemorrhagic etiology in three (21.4%) and thrombotic in one (7.1%) patient. Other causes were infections in six cases, heart failure in three cases, and a cerebral tumor in one case. Death was related to the bridging therapy (OR:3.3 [1.1–9.9]).

## Discussion

This study offers real-world data on periprocedural DOAC management and outcomes in 901 patients (53.5% major surgery). It has shown low complication rates (1.6% of thrombotic events and 1.9% of major bleeding events), even in patients who underwent moderate to high bleeding risk procedures.

Recommendations on DOAC management during the perioperative period (time of interruption before surgery and resumption afterward) are mainly based on their pharmacokinetics, renal function, and periprocedural-associated bleeding and thrombotic risk. 2 3 4 5 9 10 It has been shown that using a structured simple management approach to DOACs as proposed in PAUSE trial 17 can decrease variability and periprocedural adverse events. But DOAC regimens from real-world experience are scarce, mainly in patients undergoing major surgery procedures. As in other safety and efficacy studies, 19 20 it is necessary to know real-world data to better define perioperative recommendations.

Short-term DOAC perioperative interruption was the most common practice in the registry, being the interval longer for dabigatran (3–5 days) than for anti-Xa (2–4 days) in patients with eGFR <50 mL min 1.73 m ^−2^ , as it is mostly proposed. The perioperative management is more homogeneous than the anticoagulant reintroduction, which showed a high variability, maybe related to bleeding risk assessment during the postoperative period. In our study, this wide range of days for reintroduction of DOAC was linked to bleeding events, highlighting that 8.4% of patients were without anticoagulation 30 days after the procedure. Another possible reason for some patients could be the absence of indication.

Preoperative bridging therapy was still used in 35% of the patients (82.9% as a prophylactic dose) with similar rates compared with the Dresden Study (29.8%), 16 reflecting the difficulties to implement the guidelines, since nowadays there is a general agreement about not bridging. Our results agree with the guidelines on the lack of thrombotic protection of bridging and on its increasing risk of periprocedural hemorrhage.

Patient-specific thrombotic risk was calculated with the CHA _2_ DS _2_ -VASc score in the case of atrial fibrillation (AF). Pooled incidence of thrombotic events was 1.6%, slightly higher than the rates previously described in a real-world setting (1% in the Dresden registry). 16 Nevertheless, considering only the ischemic stroke incidence (0.6%), our results agree with the ones described in phase III DOAC studies (0.31–0.49%). They also agree with the recently published PAUSE study, with an incidence between 0.16 and 0.6 depending on the cohort. 17 Recommendations made in 2018 to 2019 propose a 1 to 3 days DOAC withdrawal time, mainly depending on DOAC type, renal function, and periprocedural hemorrhagic risk. 2 9 21 This is the first study that shows an association between preoperative DOAC withdrawal time longer than 3 days and thrombotic events, increasing as this time is longer.

Interestingly, not only preoperative DOAC withdrawal time was an independent factor for thrombotic events, but also DOAC-free time longer than 6 days. Both underlie the importance of an adequate and no-long window without anticoagulation, before and after surgery. Certainly, many patients received thromboprophylaxis with LMWH (55.3%) till the DOAC restart, but this does not mean to start over anticoagulation.

Our results showed that dabigatran was associated with a significantly higher rate of minor bleeding (12.8%) than that related to the other DOACs (apixaban 7.8%, rivaroxaban 5.7%, *p* = 0.008). It was higher but not comparable with the results of the RELY trial subanalysis (8.5% of minor bleeding events), 12 in which less than 10% of patients underwent high-bleeding risk procedures.

Pooled rate of bleeding events was 10.4%, with 8.5% of the events considered as minor and 1.9% (17/901) considered as major bleeding, but the comparison with other reports is difficult. In a recent meta-analysis, Shaw et al 11 published data on the safety in DOACs management in the perioperative period, with few data related to high bleeding risk procedures, thus, not reflecting real world. 22 A standardized protocol, as used in the PAUSE study, implied a major bleeding event rate of 0.9to 1.35 to 1.85% (dabigatran–apixaban–rivaroxaban cohort, respectively) considering less than 35% of high bleeding risk procedures. 17

No spinal bleeding was found. Nevertheless, having performed 229/901 neuraxial techniques, there is not enough statistical power to draw any real conclusion or recommendation related to the preoperative withdrawal time of the DOAC to perform a safe neuraxial puncture.

An important result to consider is that minor (OR: 2.1) and major (OR: 4.68) bleeding events were related to the use of preoperative bridging therapy. Our data collection began in late 2014, when its use was recommended in high thrombotic risk patients, 6 7 8 but bridging was also registered in the last recruitment year, against current guidelines recommendation. 5 9 21 22 23 These results are similar to the Dresden registry. In 2015, the BRIDGE trial related bridging to a higher rate of bleeding in patients receiving VKAs, 24 but the recommendations at that time were not conclusive for DOACs. 25 In any case, our results support recommendations against the use of bridging therapy in periprocedural DOACs management, agreeing that it is overprescribed. 21

One patient died during the 30-day follow-up from complications of a thrombotic etiology and three patients from bleeding causes. When considering all deaths, again bridging therapy and DOAC-free time had a significant impact on risk of death. Nevertheless, the mortality rate is too small to draw conclusions and these data should be confirmed in larger studies.

### Weaknesses in the Study

The main real-world study limitations are due to its observational characteristics and lack of randomization. Nevertheless, before performing statistics, we checked each group basal characteristics, finding that there was no significant difference related to baseline characteristics.

Most of the patients included in the study had AF (97%). Nevertheless, patients with VTE were not excluded, since we considered they are value for general measurements. But further studies are necessary to confirm the results in patients on DOAC without FA. In the same line, we did not exclude patients on edoxaban, although they could not be studied as a different drug.

The assessment of the thrombotic risk was not related to the procedure, nor was the Caprini score collected. However, considering that the patients in the study are on chronic anticoagulant treatment, that is secondary prophylaxis, the recommendation would be pharmacologic prophylaxis for all of them. 26

In line with other studies results we have found low number of events (thrombotic, major bleeding, death). But, given the observational nature of the study, there is a chance that variables not considered in the study could have influenced some of the results, such as the postoperative LMWH therapy. Besides, minor bleeding assessment, as defined in the study, can be a concern since it may be considered as subjective.

This study was performed in a wide variety of hospitals with different periprocedural DOACs management. Furthermore, thrombotic and bleeding events were needed to be clinically relevant to be considered. Despite this, we believe that the remote continuous auditing and training of the investigators, support the quality of the data collected.

### Future Studies

Although a randomized controlled trial (RCT) could be recommended to standardized practice, the PAUSE trial results represent good data in the field of periprocedural DOACs, and an RCT is unlikely to take place if the results of PAUSE are well adopted.

## Conclusion

RA-ACOD is a real-world registry performed with the aim of evaluating periprocedural thrombotic and bleeding events in unselected patients on DOAC chronic anticoagulation, scheduled for surgery or invasive procedures. Our results, with a pooled rate of all thrombotic (1.6%) and major bleeding events (1.9%), showed that the perioperative DOACs management in current clinical practice is associated with a low complication rate.

Since a long preoperative DOAC withdrawal and DOAC-free time were related to a higher incidence of thrombotic events, and the use of bridging therapy to a higher incidence of bleeding events, we would suggest a short preprocedural DOAC interruption, without bridging therapy and a reduced DOAC-free time, as a safe and suitable practice.

## References

[1] Eikelboom J W Weitz J I New anticoagulants Circulation 2010 121 13 1523 1532 20368532 10.1161/CIRCULATIONAHA.109.853119

[2] Spahn D R Beer J H Borgeat A NOACs in anesthesiology Transfus Med Hemother 2019 46 04 282 293 31700511 10.1159/000491400 PMC6739714

[3] Douketis J D Spyropoulos A C Spencer F A Perioperative management of antithrombotic therapy Chest 2012 141 02 e326S e350S 22315266 10.1378/chest.11-2298 PMC3278059

[4] Keeling D Tait R C Watson H; British Committee of Standards for Haematology.Peri-operative management of anticoagulation and antiplatelet therapy Br J Haematol 2016 175 04 602 613 27714755 10.1111/bjh.14344

[5] Kozek-Langenecker S A Ahmed A B Afshari A Management of severe perioperative bleeding: guidelines from the European Society of Anaesthesiology: first update 2016 Eur J Anaesthesiol 2017 34 06 332 395 28459785 10.1097/EJA.0000000000000630

[6] Ferrandis R Castillo J de Andrés J The perioperative management of new direct oral anticoagulants: a question without answers Thromb Haemost 2013 110 03 515 522 23846475 10.1160/TH12-11-0868

[7] Kozek-Langenecker S A Afshari A Albaladejo P Management of severe perioperative bleeding: guidelines from the European Society of Anaesthesiology Eur J Anaesthesiol 2013 30 06 270 382 23656742 10.1097/EJA.0b013e32835f4d5b

[8] Sié P Samama C M Godier A Surgery and invasive procedures in patients on long-term treatment with direct oral anticoagulants: thrombin or factor-Xa inhibitors. Recommendations of the Working Group on Perioperative Haemostasis and the French Study Group on Thrombosis and Haemostasis Arch Cardiovasc Dis 2011 104 12 669 676 22152517 10.1016/j.acvd.2011.09.001

[9] Vivas D Roldán I Ferrandis R Perioperative and periprocedural management of antithrombotic therapy: consensus document of SEC, SEDAR, SEACV, SECTCV, AEC, SECPRE, SEPD, SEGO, SEHH, SETH, SEMERGEN, SEMFYC, SEMG, SEMICYUC, SEMI, SEMES, SEPAR, SENEC, SEO, SEPA, SERVEI, SECOT and AEU Rev Esp Cardiol (Engl Ed) 2018 71 07 553 564 29887180 10.1016/j.rec.2018.01.029

[10] Steffel J Verhamme P Potpara T S The 2018 European Heart Rhythm Association Practical Guide on the use of non-vitamin K antagonist oral anticoagulants in patients with atrial fibrillation Eur Heart J 2018 39 16 1330 1393 29562325 10.1093/eurheartj/ehy136

[11] Shaw J R Woodfine J D Douketis J Schulman S Carrier M Perioperative interruption of direct oral anticoagulants in patients with atrial fibrillation: a systematic review and meta-analysis Res Pract Thromb Haemost 2018 2 02 282 290 30046730 10.1002/rth2.12076 PMC6055497

[12] Healey J S Eikelboom J Douketis J Periprocedural bleeding and thromboembolic events with dabigatran compared with warfarin: results from the Randomized Evaluation of Long-Term Anticoagulation Therapy (RE-LY) randomized trial Circulation 2012 126 03 343 348 22700854 10.1161/CIRCULATIONAHA.111.090464

[13] Sherwood M W Douketis J D Patel M R Outcomes of temporary interruption of rivaroxaban compared with warfarin in patients with nonvalvular atrial fibrillation: results from the rivaroxaban once daily, oral, direct factor Xa inhibition compared with vitamin K antagonism for prevention of stroke and embolism trial in atrial fibrillation (ROCKET AF) Circulation 2014 129 18 1850 1859 24552831 10.1161/CIRCULATIONAHA.113.005754 PMC4206548

[14] Garcia D Alexander J H Wallentin L Management and clinical outcomes in patients treated with apixaban vs warfarin undergoing procedures Blood 2014 124 25 3692 3698 25320240 10.1182/blood-2014-08-595496 PMC4263979

[15] Douketis J D Weitz J I Murphy S Deenadayalu N Crompton A E Perioperative adverse outcomes in patients with atrial fibrillation taking edoxaban or warfarin: analysis of the ENGAGE AF-TIMI 48 trial J Am Coll Cardiol 2015 65 10 A2092

[16] Beyer-Westendorf J Gelbricht V Förster K Peri-interventional management of novel oral anticoagulants in daily care: results from the prospective Dresden NOAC registry Eur Heart J 2014 35 28 1888 1896 24394381 10.1093/eurheartj/eht557

[17] Douketis J D Spyropoulos A C Duncan J Perioperative management of patients with atrial fibrillation receiving a direct oral anticoagulant JAMA Intern Med 2019 179 11 1469 1478 10.1001/jamainternmed.2019.2431 PMC6686768 31380891

[18] Schulman S Angerås U Bergqvist D Eriksson B Lassen M R Fisher W; Subcommittee on Control of Anticoagulation of the Scientific and Standardization Committee of the International Society on Thrombosis and Haemostasis.Definition of major bleeding in clinical investigations of antihemostatic medicinal products in surgical patients J Thromb Haemost 2010 8 01 202 204 19878532 10.1111/j.1538-7836.2009.03678.x

[19] Jun M Lix L M Durand M Comparative safety of direct oral anticoagulants and warfarin in venous thromboembolism: multicentre, population based, observational study BMJ 2017 359 j4323 29042362 10.1136/bmj.j4323 PMC5641962

[20] Vinogradova Y Coupland C Hill T Hippisley-Cox J Risks and benefits of direct oral anticoagulants versus warfarin in a real world setting: cohort study in primary care BMJ 2018 362 k2505 29973392 10.1136/bmj.k2505 PMC6031213

[21] McIlmoyle K Tran H Perioperative management of oral anticoagulation BJA Educ 2018 18 09 259 264 10.1016/j.bjae.2018.05.007 PMC7808108 33456842

[22] Lijfering W M Tichelaar Y IGV Direct oral anticoagulant use and risk of perioperative bleeding: evidence of absence or absence of evidence? Res Pract Thromb Haemost 2018 2 02 182 185 30046719 10.1002/rth2.12084 PMC6055550

[23] Tafur A Douketis J Perioperative management of anticoagulant and antiplatelet therapy Heart 2018 104 17 1461 1467 29217632 10.1136/heartjnl-2016-310581

[24] Douketis J D Spyropoulos A C Kaatz S Perioperative bridging anticoagulation in patients with atrial fibrillation N Engl J Med 2015 373 09 823 833 26095867 10.1056/NEJMoa1501035 PMC4931686

[25] Daniels P R Peri-procedural management of patients taking oral anticoagulants BMJ 2015 351 h2391 26174061 10.1136/bmj.h2391

[26] Chee Y L Crawford J C Watson H G Greaves M; British Committee for Standards in Haematology.Guidelines on the assessment of bleeding risk prior to surgery or invasive procedures Br J Haematol 2008 140 05 496 504 18275427 10.1111/j.1365-2141.2007.06968.x

