# Supplementary material for: Periprocedural Direct Oral Anticoagulant Management: The RA-ACOD Prospective, Multicenter Real-World Registry
Source: TH Open. 2020 Jun 26;4(2):e127–37. doi: 10.1055/s-0040-1712476 (PMC7319799; doi:10.1055/s-0040-1712476)
Supplement: Supplementary file 1 — Supplementary Material [file 10-1055-s-0040-1712476-s200009.pdf]

Supplementary Appendix: RA-ACOD Investigators

- Clínica Universidad de Navarra: Francisco Hidalgo, Juan A. Martínez.
- Complejo Hospitalario de Toledo: Beatriz Castaño.
- Fundació Puigvert: Pilar Sierra, Diana Vernetta, Ana Álvarez, Sergi Sabaté.
- Hospital Dr Peset: Julia Martín, Estefania Martínez, Joana Baldó.
- Hospital Clínic Universitari de València: Juan V. Llau, Raquel Ferrandis, Concepción Rubio, Ana Izquierdo, José Juste.
- Hospital Costa del Sol (Marbella): Manuel Llácer, Antonio Vivó, Rafael García,
- Hospital de Galdakao-Usánsolo: Óscar González, Pilar Romero, Marta Errazquinaguirre, Elisa Díez, Carlos Santos.
- Hospital General Universitario Gregorio Marañón: F. Javier Sanz, Francisco González, Guillermo Sánchez, Ignacio Garutti, María Lema, Luis Olmedilla.
- Hospital Lluís Alcanyís de Xàtiva: Esperanza Fernández, Vicente Domingo.
- Hospital de Mataró (Consorci Sanitari del Maresme): Antonio Pérez.
- Hospital de Manises: M. Consuelo García, Juan R. De Moya, Eva Romero, Estrella M. Domenech.
- Hospital Miguel Servet: M. Concepción Cassinello, M. Pilar Herranz, Leticia Oliden, Ana Moleda.
- Hospital La Paz: Blanca Tapia.
- Hospital Puerta del Mar: Luis Miguel Torres, Eduardo de la Concha, María de las Montañas González.
- Hospital de Sabadell-Parc Taulí: Marta Barquero, Helena Mendez, Laura Ricol, Andrea Vallejo.
- Hospital Universitario de Canarias: Nuria Montón, M. Carmen Martín, Vanesa González.
- Hospital Universitario de Getafe: Elena Duro.
- Hospital Universitario de Gran Canaria Dr. Negrín: Vanessa Suárez, Maria Luisa Torres, Javier García, Nazario Ojeda, Aurelio Rodríguez.
- Hospital Universitario Marqués de Valdecilla: M. Ángeles Villanueva.
- Hospital Universitario de Móstoles: Rubén Rodríguez, Raquel Fernández.
- Hospital Universitari i Politècnic La Fé: M. Salomé Matoses, Natalia García, Jaime López, Sandra Verdeguer.
- Hospital Universitario La Ribera: Cristina Martínez, Sonsoles Aragón Álvarez, Vicente Muedra.
- Hospital Universitario Parc de Salut Mar: Juan Carlos Álvarez García.
- Hospital Universitario Vall d’Hebron: Patricia Guilabert, Nuria Martín, Luis Abarca, Gemma Maria Usúa.
- Hospital Universitario Virgen del Rocío: Gabriel José Yanes, Emilio José Guerrero.
- Hospital Virgen de la Victoria: J. Aurelio Gómez-Luque, José Luís Guerrero.

Supplementary Material S1 Definitions

Estimated Glomerular Filtration Rate

It was calculated using the Cockcroft-Gault formula:

CrCl (male) = ([140-age] × weight in kg)/(serum creatinine × 72)
CrCl (female) = ([140-age] × weight in kg × 0.85)/(serum creatinine × 72)

References

National Kidney Foundation. Available at: https://www.kidney.org/professionals/KDOQI/gfr. Accessed February 10, 2014

Cockcroft DW, Gault MH. Prediction of creatinine clearance from serum creatinine. Nephron 1976;16(1):31–41

Thrombotic Risk

For the classification of thrombotic risk, the CHA_2DS_2-VASc score in the case of atrial fibrillation or the time since venous thromboembolism with or without associated thrombophilia was used. Patients were classified as at low, moderate, or high thrombotic risk.

Atrial fibrillation: CHA2DS2-VASc score

Risk factor	Score
Congestive heart failure or left ventricular disfunction	1
Hypertension	1
Age ≥75 y	2
Diabetes mellitus	1
Stroke or transient ischemic attack or thromboembolism	2
Vascular disease	1
Age 65–74 y	1
Sex category (female gender)	1

Note: Low thrombotic risk: score 0–4; moderate thrombotic risk: score 5–6; high thrombotic risk: score 7–9.

Venous thromboembolism

Low	• Venous thromboembolism >12 mo.
Moderate	• Venous thromboembolism 3–12 mo.<br>• Moderate thrombophilia.
High	• Venous thromboembolism <3 mo.<br>• Severe thrombophilia.

References

Douketis JD, Spyropoulos AC, Spencer FA, et al. Perioperative management of antithrombotic therapy: antithrombotic therapy and prevention of thrombosis, 9th ed: American College of Chest Physicians evidence-based clinical practice guidelines. Chest 2012;141(suppl 2):e326S–e350S

Lip GYH, Nieuwlaat R, Pisters R, Lane DA, Crijns HJGM; The Euro Heart Survey on Atrial Fibrillation. Refining clinical risk stratification for predicting stroke and thromboembolism in atrial fibrillation using a novel risk factor-based approach: the euro heart survey on atrial fibrillation. Chest 2010;137 (2):263–272

Bleeding Risk

The classification of perioperative/periprocedural bleeding risk was based on the hemorrhagic risk classification according to the surgery proposed by the Spanish Forum on Anticoagulants and Anesthesia and finally assessed by the team in charge (anesthesiologist and surgeon) based on the type of surgery, the team experience, and the characteristics of the patient.

Bleeding risk classification

Low	• If necessary, appropriate hemostasis can be achieved.<br>• A possible bleeding does not expose the patient to a vital risk nor put at risk the surgery outcome.<br>• No transfusion is usually needed.<br>• Examples: minor surgery (plastic, minor orthopaedics, endoscopic ear, nose and throat surgery, eye anterior chamber surgery, dental procedures).
Moderate	• If necessary, surgical hemostasis can be difficult.<br>• A possible bleeding increases the need of transfusion or it implies a need of reintervention.<br>• Examples: major abdominal surgery, cardiovascular, major orthopaedics, ear, nose and throat, urology, reconstructive.
High	• A perioperative bleeding may put at risk the patient life or the surgery outcome.<br>• Examples: intracranial neurosurgery, intervention in the spinal cord, eye posterior chamber surgery.

Reference

Llau JV, Ferrandis R, Castillo J, et al. participantes en el Foro de Consenso de la ESRA-España sobre «Fármacos que alteran la hemostasia». Management of oral direct anticoagulants in the perioperative period and invasive techniques. Rev Esp Anestesiol Reanim 2012;59:321–330

Bridging Therapy

Bridging therapy was defined as the administration of a short-acting anticoagulant, such as a low-molecular-weight heparin or unfractionated heparin, during the time when the direct oral anticoagulant was being withheld before the surgery (preoperative bridging therapy) and subsequently

after the surgery or the invasive procedure until the direct oral anticoagulant was resumed (postoperative bridging therapy). In this registry all patients with a bridging therapy protocol were under a low-molecularweight heparin.

Reference

Ortel TL. Perioperative management of patients on chronic antithrombotic therapy. Blood 2012;120(24):4699–4705

DOAC-Free Time

DOAC-free time was defined as the time in which the patient was without receiving the direct oral anticoagulant: time since preoperative withdrawal till the postoperative restart.

Thrombotic Event

Thrombotic events were defined following the recommendations of the European Society of Anaesthesiology (ESA) and European Society of Intensive Care Medicine (ESCIM) joint taskforce on perioperative outcome measures:

- *Acute coronary syndrome*: Increase in serum cardiac troponin with at least one value above the 99th percentile upper reference limit, with or without ECG changes.
- *Stroke*: any embolic, thrombotic, or hemorrhagic cerebral event with persistent residual motor, sensory, or cognitive dysfunction (e.g., hemiplegia, hemiparesis, aphasia, sensory deficit, impaired memory).
- *Transient ischemic attack*: any nonpersistent stroke, with full motor, sensory, or cognitive recovery.
- *Venous thromboembolism*: symptomatic deep vein thrombosis and/or pulmonary embolism clinically suspected and confirmed by appropriate diagnostic tests following local protocol that could include ultrasound, venography, computed tomography or magnetic resonance imaging venography.
- *Arterial embolism*: diagnosis of a blood clot or thrombus within the arterial system.

Reference

Jammer I, Wickboldt N, Sander M, et al; European Society of Anaesthesiology (ESA) and the European Society of Intensive Care Medicine (ESICM); European Society of Anaesthesiology; European Society of Intensive Care Medicine. Standards for definitions and use of outcome measures for clinical effectiveness research in perioperative medicine: European Perioperative Clinical Outcome (EPCO) definitions: a statement from the ESA-ESICM joint taskforce on perioperative outcome measures. Eur J Anaesthesiol 2015;32(2):88–105

Bleeding Event

Bleeding events were classified as *major* or *minor*.

Definition of *major bleeding* was based on the Subcommittee on Control of Anticoagulation of the Scientific and Standardization Committee of the International Society on Thrombosis and Haemostasis proposals:

- Any fatal hemorrhage, or
- Any bleeding related to a decrease in hemoglobin $>$ 2 g/dL, or
- Any bleeding requiring reintervention, or
- Any symptomatic bleeding in a critical area or organ.

Definition of *minor bleeding* was:

- Any hemorrhagic event not classified as major.

Reference

Schulman S, Angerås U, Bergqvist D, Eriksson B, Lassen MR, Fisher W; Subcommittee on Control of Anticoagulation of the Scientific and Standardization Committee of the International Society on Thrombosis and Haemostasis. Definition of major bleeding in clinical investigations of antihemostatic medicinal products in surgical patients. J Thromb Haemost 2010;8(1):202–204

Supplementary Material S2 Patients Who Did Not Reintroduce DOAC in the 30 Days Follow-up

Seventy-seven patients did not have the DOACs reintroduced (37 on apixaban, 15 on dabigatran, 24 on rivaroxaban, and one on edoxaban). The patient's thrombotic risk was low in 46, moderate in 27, and high in 4 cases. The hemorrhagic risk was low in 17, moderate in 42, and high in 16 patients. Preoperative bridging therapy was prescribed in 49 patients (31 as prophylactic dose and 18 as therapeutic dose).

No postoperative bridging therapy was prescribed in nine patients, with only six of them having mechanical prophylaxis. In three cases the patients died, one due to a major hemorrhagic event, that required reoperation (he was with intermittent pneumatic compression), and the others to a septic shock and a cerebral tumor. We have no explanation for the other six.

Postoperative bridging therapy with a prophylactic dose was prescribed in 46 patients (with concomitant mechanical prophylaxis in ten cases). Four patients died, one due to a major hemorrhagic event, and two due to a septic shock. Other three patients suffered a major hemorrhagic event and three patients a minor hemorrhagic event. In three cases a new intervention was needed (two cases due to infection). ACOD was substituted by acenocoumarol in one patient. We have no explanation for the 31 remaining patients.

Postoperative bridging therapy with a therapeutic dose was prescribed in 22 patients (with concomitant mechanical prophylaxis in two cases). Three patients died, one due to a major hemorrhagic event (in a second intervention not related to ACOD management, see ►**Supplementary Material Table S6**), one due to a stroke, and one due to a complicated pneumonia. Other two patients suffered a major hemorrhagic event and eight patients a minor hemorrhagic event. We have no explanation for the nine remaining patients.

Supplementary Table S1 Variables and data collection

General data	
	Demographics and patient history<br>DOAC therapy<br>Classification of thrombotic risk (low–moderate–high) based on the CHA_2DS_2-VASc score in the case of atrial fibrillation[9] or on the time since venous thromboembolism<br>Classification of bleeding risk (low–moderate–high) based on the type of surgery[a]<br>Renal function: serum creatinine and eGFR (estimated glomerular filtration rate)<br>Type of surgery[b]<br>Anesthesia management
Perioperative anticoagulant management	
	Time of DOAC withdrawal (before surgery)<br>Time to DOAC restart (after surgery)<br>DOAC-free time: time of withdrawal plus time of DOAC restart<br>Use of preoperative bridging therapy<br>Postoperative thromboprophylaxis:<br>• Pharmacological (LMWH)<br>• Mechanical thromboprophylaxis
Thrombotic events	
	Acute coronary syndrome<br>Stroke<br>Transient ischemic attack<br>Venous thromboembolism: symptomatic deep vein thrombosis, pulmonary embolism<br>Arterial embolism
Bleeding events	
	Subjective intraoperative bleeding assessment<br>Need for transfusion or hemostatics<br>Need for surgical revision due to bleeding
Death	Cause of death

Abbreviations: DOAC, direct oral anticoagulant; LMWH, low-molecular weight heparin.
[a]See reference 13.
[b]See reference 10.

Supplementary Table S2 Hospitals characteristics and local protocols

Hospital	Number of beds	Number of operating theater	Protocol for DOAC management	Preoperative bridging therapy	LMWH	Preoperative LMWH dose	Postoperative thromboprophylaxis
Clínica U. de Navarra	240	18	Yes	Yes	Bemiparin	Prophylactic	Multidisciplinary local protocol
Clínic U. València	600	15	Yes	No	Bemiparin Enoxaparin	–	Multidisciplinary local protocol
C. Hospitalario de Toledo	635	27	Yes	Yes	Enoxaparin	Therapeutic	Individual decision
Costa del Sol de Marbella	408	10	No	Yes	Enoxaparin	Treatment	Surgeons protocol
Doctor Peset	531	21	No	No	Bemiparin	–	Surgeons protocol
Fundació Puigvert	150	4	Yes	No	Bemiparin	–	LMWH bridging
Galdakao-Usansolo	400	16	Yes	Yes	Bemiparin Enoxaparin	Prophylactic	Multidisciplinary local protocol
General U. Gregorio Marañón	1,351	44	Yes	No	Bemiparin Enoxaparin	–	Multidisciplinary local protocol
Lluís Alcanyís de Xàtiva	230	8	Yes	Yes	Enoxaparin	Prophylactic	Surgeons protocol
Manises	300	10	Yes	Yes	Enoxaparin	Both	Multidisciplinary local protocol
Mataró	250	8	Yes	Yes	Bemiparin	Prophylactic	Multidisciplinary local protocol
Miguel Servet	1,311	28	Yes	Yes	Bemiparin Enoxaparin	Prophylactic	Multidisciplinary local protocol
Parc Salut Mar	500	19	Yes	No	Bemiparin	–	Multidisciplinary local protocol
Puerta del Mar	700	20	Yes	Yes	Enoxaparin	Both	Multidisciplinary local protocol
Sabadell-Parc Taulí	800	16	Yes	No	Bemiparin Enoxaparin	–	LMWH bridging
U. de Canarias	655	21	Yes	Yes	Enoxaparin	Both	Surgeons protocol
U. de Getafe	524	14	Yes	Yes	Enoxaparin	Both	Surgeons protocol
U. Gran Canaria Dr. Negrín	629	20	Yes	Yes	Enoxaparin	Prophylactic	Multidisciplinary local protocol
U. La Paz	1,254	50	Yes	Yes	Enoxaparin	Both	Multidisciplinary local protocol
U. Marqués de Valdecillas	910	24	Yes	No	–	–	Surgeons protocol
U. Móstoles	294	12	No	No	–	–	Prophylactic LMWH
U. i Politècnic La Fe	1,000	40	Yes	Yes	Bemiparin Enoxaparin	Both	Multidisciplinary local protocol
U. La Ribera	300	13	Yes	Yes	Bemiparin	Prophylactic	Individual decision
U. Vall d'Hebrón	234	13	No	Yes	Enoxaparin	Prophylactic	Surgeons protocol
U. Virgen del Rocio	1,279	51	Yes	Yes	Enoxaparin	Prophylactic	Multidisciplinary local protocol
Virgen de la Victoria	542	17	Yes	Yes	Enoxaparin	Therapeutic	Multidisciplinary local protocol

Abbreviations: C, Complejo; DOAC, direct oral anticoagulant; LMWH, low-molecular weight heparin; U, Universitario.

Supplementary Table S3 Characteristics of patients and procedures compared by the type of DOAC

	All DOAC	Dabigatran	Apixaban	Rivaroxaban	Edoxaban
	n = 901	*n* = 267	*n* = 322	*n* = 304	*n* = 8
Demographic data					
Gender, male [n (%)]	553 (61.4)	167 (62.5)	205 (63.7)	175 (57.6)	6 (75)
Age [y, median (25–75%)]	75 (69–81)	74 (68–79.8)	78 (71–82)	75 (68–80)	67 (64–68)
Weight [kg, median (25–75%)]	79 (70–89)	80 (70–86)	77 (65.5–89.5)	80 (70–90)	78 (66–98)
Height [cm, median (25–75%)]	165 (159–172)	165 (159–171)	165 (159–172)	165 (159–172)	167 (150–170)
eGFR [median (25–75%)]	77.9 (58.4–101.3)	80.9 (66.1–99.1)	70.6 (54.1–100.2)	80.3 (58.4–104.6)	67.1 (56.2–79.1)
Comorbidity [*n* (%)]					
History of heart failure	81 (9.0)	21 (7.9)	40 (12.4)	20 (6.6)	0 (0)
Hypertension	731 (81.1)	225 (84.3)	264 (82)	236 (77.6)	6 (75)
Diabetes mellitus	284 (31.5)	84 (31.5)	102 (31.7)	95 (31.3)	3 (37.5)
Stroke	200 (22.2)	64 (24)	83(25.8)	51 (16.8)	2 (25)
Pulmonary embolism	14 (1.6)	4 (1.5)	2 (0.6)	8 (2.6)	0 (0)
Deep vein thrombosis	33 (3.7)	6 (2.2)	4 (1.2)	23 (7.6)	0 (0)
Peripheral artery disease	178 (19.8)	47 (17.6)	74 (23)	56 (18.4)	1 (12.5)
Active oncologic disease	173 (19.2)	49 (18.4)	71 (22)	52 (17.1)	1 (12.5)
Thrombophilia	8 (0.9)	1 (0.4)	3 (0.9)	4 (1.3)	0 (0)
Liver disease	36 (4.0)	9 (3.4)	17 (5.3)	10 (3.3)	0 (0)
Alcohol abuse	73 (8.2)	20 (7.5)	23 (7.1)	28 (9.2)	2 (25)
Street drug addiction	3 (0.3)	0 (0)	1 (0.3)	2 (0.7)	0 (0)
DOAC indication [*n* (%)]					
Atrial fibrillation	874 (97.0)	263 (98.5)	316 (98.1)	287 (94.4)	8 (100)
Venous thromboembolism	15 (1.7)	1 (0.4)	3 (0.9)	11 (3.6)	0 (0)
Recurrent venous thromboembolism	12 (1.3)	3 (1.1)	3 (0.9)	6 (2.0)	0 (0)
Concomitant treatment [*n* (%)]					
Aspirin	77 (8.5)	24 (9)	24 (7.5)	29 (9.5)	0 (0)
Clopidogrel	11 (1.2)	4 (1.5)	4 (1.2)	3 (1)	0 (0)
NSAID	13 (1.4)	3 (1.1)	4 (1.2)	6 (2)	0 (0)
Amiodarone	80 (8.9)	27 (10.1)	27 (8.4)	25 (8.2)	1 (12.5)
Verapamil	7 (0.8)	3 (1.1)	3 (0.9)	1 (0.3)	0 (0)
Ketoconazole	1 (0.1)	0 (0)	0 (0)	1 (0.3)	0 (0)
Dronedarone	7 (0.8)	2 (0.7)	1(0.3)	4 (1.3)	0 (0)
Other *p*-glycoprotein inhibitors	54 (6.0)	17 (6.4)	19 (5.9)	16 (5.3)	2 (25)
Patient thrombotic risk[a] [*n* (%)]					
Low	619 (68.7)	188 (70.4)	210 (65.2)	213 (70.1)	8 (100)
Moderate	227 (25.2)	62 (23.2)	85 (26.4)	80 (26.3)	0 (0)
High	55 (6.1)	17 (6.4)	27 (8.4)	11 (3.6)	0 (0)

(*Continued*)

Supplementary Table S3 (*Continued*)

	All DOAC	Dabigatran	Apixaban	Rivaroxaban	Edoxaban
	n = 901	*n* = 267	*n* = 322	*n* = 304	*n* = 8
Bleeding risk of procedurea [*n* (%)]					
Low	412 (45.7)	118 (44.2)	160 (49.7)	130 (42.8)	4 (50)
Moderate	427 (47.4)	126 (47.2)	140 (43.5)	157 (51.6)	4 (50)
High	62 (6.9)	23 (8.6)	22 (6.8)	17 (5.6)	0 (0)
Type of anaesthesia [*n* (%)]					
General	515 (57.0)	142 (53.2)	189 (58.7)	178 (58.6)	6 (75)
Neuraxial block	229 (25.5)	73 (27.3)	76 (23.6)	80 (26.3)	0 (0)
Nerve block	72 (8.0)	18 (6.7)	29 (9)	23 (7.6)	2 (25)
Local	85 (9.5)	34 (12.7)	28 (8.7)	23 (.6)	0 (0)
CHA2DS2-VASc score [*n* (%)]					
Subjects (*n*)[b]	874	263	316	287	8
0–4	613 (67.5)	188 (71.5)	209 (66.1)	208 (72.3)	8 (100)
5–6	212 (24.3)	59 (22.4)	82 (26.0)	71 (24.7)	0
7–9	49 (5.6)	16 (6.1)	25 (7.9)	8 (2.8)	0

Abbreviations: DOAC, direct oral anticoagulant; eGFR, estimated glomerular filtration rate; NSAID: non-steroid anti-inflammatory drug; CHA_2DS_2-VASc score,1 point for congestive heart failure, hypertension, diabetes mellitus, female, age 65–74 y and vascular disease (peripheral artery disease, myocardial infarction, aortic plaque); 2 points for age ≥75 y, prior stroke or transient ischemic attack or thromboembolism.
[a]See ►**Supplementary Material S1** and ►**Supplementary Table S4**.
[b]Only patients with Atrial Fibrillation.

Supplementary Table S4 Detailed type of surgery and related estimated bleeding risk

General type of surgery *n* (%)	Detailed type of surgery	Estimated bleeding risk		
		Low (*n*)	Moderate (*n*)	High (*n*)
Minor surgery 235 (26.1)	Urologic			
	Prostate biopsy	4	1	
	Urethroscopy/urethrostomy	8	1	
	Double J stent	8	2	
	Ureteroscopy	1	4	
	Other	2	8	
	General surgery			
	Minor abdominal wall surgery	21	9	
	Hemorrhoidectomy	2	3	
	Other	2	2	
	Orthopaedic			
	Minor hand/foot surgery	23	2	
	Arthroscopy	5	2	
	Other	6	3	
	Dermatologic surgery	47	8	
	Peripheral minor vascular	2	4	
	Cardiac/Vascular ablation	24	12	
	Pacemaker	2	2	
	Maxillofacial surgery	15	2	
Major nonorthopaedic 304 (33.7)	Urologic			
	TUR bladder	12	40	
	TUR prostate	1	20	2
	Nephro/urologic neoplasm		11	
	Prostatectomy		7	
	Urethral repair surgery	4	2	
	Other	8	4	
	General surgery			
	Major abdominal wall surgery	8	5	
	Cholecystectomy	10	17	4
	Open abdominal neoplasm		25	
	Laparoscopic neoplasm		13	
	Other	2	4	
	Major cardiovascular			
	Angioplasty/endarterectomy	3	14	8
	Cardiac surgery		8	9
	Other major vascular surgery	1	3	
	Thoracic surgery	3	14	2
	Neurosurgery		1	4
	ENT-neoplasm	7	14	1
	Gynecological surgery	5	8	
Major orthopaedic 178 (19.8)	Total hip arthroplasty		32	
	Total knee arthroplasty		48	5

(*Continued*)

Supplementary Table S4 (*Continued*)

General type of surgery *n* (%)	Detailed type of surgery	Estimated bleeding risk		
		Low (*n*)	Moderate (*n*)	High (*n*)
	Hip fracture		19	1
	Upper limb major surgery	7	8	
	Lower limb major surgery	13	11	
	Spine surgery	2	4	14
	Other major surgery	6	7	1
Endoscopy 51 (5.7)	Colonoscopy and/or gastroscopy	46	5	
Ophthalmic surgery 37 (4.1%)	Cataract	47		
	Vitrectomy/retina surgery	2	1	11
	Phacoemulsification	12		
	Ectropion surgery	6		
	Other ophthalmic surgery	13	4	
Other 37 (4.1%)	Miscellany	22	15	
Total ($n = 901$)		412	427	62
Total (%)		(45.7%)	(47.4%)	(6.9%)

Abbreviations: ENT, ear, nose, and throat; TUR, transurethral resection.
Note: The bleeding risk was estimated from the type of surgery and the specific characteristics of the patients. So, the same kind of procedure could have different estimated bleeding risk.

Supplementary Table S5 Detailed characteristics of patients suffering a thrombotic event

ID	Age (y)	Gender	BMI	DOAC indication	DOAC dose	Type of surgery	Thrombotic risk[a] (CHA_2DS_2-VASc score)	Type of thrombotic event	Surgery bleeding risk[b]	Bleeding event[a]	Preoperative DOAC withdrawal time (d)	Preoperative LMWH (dose)	Postoperative DOAC restart time (d)[c]	Postoperative LMWH (dose)
125	67	Female	40	AF	Dabigatran 150 mg/12 h	Thoracic arthrodesis	Low (2)	VTE	High	No	5	Yes (Prophylactic)	No restart	Yes (Prophylactic)
192	74	Male	29	AF	Rivaroxaban 20mg/24h	Aortic endoprosthesis	Low (4)	VTE	Low	No	2	No	2	Yes (Therapeutic)
222	51	Male	34	AF	Dabigatran 150mg/12h	Shoulder arthroscopy	Low (1)	ACS	Low	No	5	No	2	No
264	75	Male	29	AF	Rivaroxaban 15mg/24h	Neumonectomy	Moderate (6)	IS	Moderate	Yes Minor	5	Yes (Prophylactic)	No restart	Yes (Prophylactic)
296	75	Male	28	AF	Dabigatran 150mg/12h	TUR prostate	Low (4)	IS	High	Yes Major	3	Yes (Prophylactic)	2	Yes (Prophylactic)
380	63	Male	30	AF	Apixaban 5mg/12h	Endoscopic urethrotomy	Low (1)	ACS	Moderate	No	5	Yes (Prophylactic)	2	No
451	83	Male	25	AF	Dabigatran 110mg/12h	Endarterectomy	High (7)	ACS	Moderate	Yes Minor	4	No	12	Yes (Therapeutic)
456	65	Male	43	AF	Apixaban 5mg/12h	Glossectomy	Low (2)	VTE	Moderate	Yes Major	2	No	No	Yes (Prophylactic)
525	80	Male	28	AF	Rivaroxaban 20mg ×1	Lobectomy	Low (3)	IS	Moderate	No	2	No	No restart	Yes (Therapeutic)
547	82	Male	34	AF	Dabigatran 110mg × 2	Colonoscopy	Low (4)	IS	Low	No	3	Yes (Therapeutic)	No restart	Yes (Therapeutic)
674	82	Female	25	AF	Apixaban 2.5mg/12h	Colectomy	Moderate (5)	ACS	Moderate	No	5	Yes (Therapeutic)	10	Yes (Prophylactic)
684	76	Male	33	AF	Apixaban 5mg/12h	Total knee arthroplasty	Low (4)	IS	Moderate	No	3	No	15	Yes (Prophylactic)
702	78	Male	31	AF	Rivaroxaban 20mg/24h	Total knee arthroplasty	High (7)	ACS	Moderate	Yes Minor	5	Yes (Therapeutic)	17	Yes (Therapeutic)
806	76	Male	35	AF	Rivaroxaban 15 mg/24h	Vitrectomy	Low (3)	ACS	High	No	2	No	1	No

Abbreviations: ACS, acute coronary syndrome; AF, atrial fibrillation; BMI, body mass index (Kg/m^2); DOAC, direct oral anticoagulant; ID, Patient identification; IS, ischemic stroke; LMWH, low molecular weight heparin, (therapeutic or prophylactic dose); TUR, transurethral resection; VTE, venous thromboembolism.

[a]See Supplementary Material S1.

[b]See ►**Supplementary Table S3**.

[c]During the 30-d follow-up.

Supplementary Table S6 Detailed characteristics of patients suffering a major bleeding event

ID	Age (y)	Gender	BMI	DOAC Indication	DOAC dose	Type of surgery	Thrombotic risk[a] (CHA_2DS_2-VASc score)	Type of thrombotic event	Surgery bleeding risk[b]	Preoperative DOAC withdrawal time (d)	Preoperative LMWH (dose)	Postoperative DOAC restart time (d)[c]	Postoperative LMWH (dose)
72	57	Male	31	AF	Dabigatran 150mg/12h	Ocular exenteration	Low (1)	No	High	5	Yes (Prophylactic)	5	Yes (Therapeutic)
131	78	Female	27	AF	Apìxaban 5mg/12h	ERCP	Moderate (6)	No	Low	3	Yes (Therapeutic)	No restart	Yes (Therapeutic)
190	72	Male	28	AF	Rivaroxaban 20mg/24h	Cordectomy (neoplasm)	Low (3)	No	Low	5	Yes (Therapeutic)	No restart	Yes (Prophylactic)
284	62	Male	24	AF	Rivaroxaban 20mg/24h	Laparoscopic cholecystectomy	Low (2)	No	Moderate	2	Yes (Prophylactic)	2	No
296	75	Male	28	AF	Dabigatran 150mg/12h	TUR prostate	Low (4)	Yes, IS	High	3	Yes (Prophylactic)	2	Yes (Prophylactic)
345	62	Male	23	AF	Rivaroxaban 20mg/24h	Laparoscopic cholecystectomy	Low (1)	No	Moderate	2	Yes (Prophylactic)	5	No
352	80	Female	24	AF	Rivaroxaban 15mg/24h	Shoulder fracture	Low (3)	No	Moderate	5	Yes (Prophylactic)	3	Yes (Prophylactic)
456	65	Male	43	AF	Apìxaban 5mg/12h	Glossectomy	Low (2)	Yes, VTE	Moderate	2	No	No restart	Yes (Prophylactic)
485	69	Female	26	AF	Rivaroxaban 20mg/24h	Cholangio-carcinoma resection	Low (2)	No	High	2	Yes (Therapeutic)	No restart	No
587	90	Female	18	AF	Apìxaban 5mg/12h	Hip fracture	High (7)	No	Moderate	6	Yes (Prophylactic)	No restart	Yes (Prophylactic)
599	80	Female	37	AF	Apixaban 2.5mg/12h	Ablation + atrium closure	Moderate (6)	No	Moderate	1	No	No restart	Yes (Prophylactic)
620	74	Male	28	AF	Dabigatran 150mg/12h	Lumbar arthrodesis	Low (2)	No	High	4	Yes (Therapeutic)	21	Yes (Prophylactic)
660	57	Female	41	VTE	Rivaroxaban 20mg/24h	Laparoscopic obesity surgery	Low (0)	No	Moderate	3	Yes (Prophylactic)	7	Yes (Prophylactic)
842	74	Male	33	AF	Apìxaban 5mg/12h	TUR bladder	Low (3)	No	Low	2	No	4	Yes (Prophylactic)
1000	86	Female	25	AF	Apixaban 2.5mg/12h	Cataract	High (9)	No	Low	1	No	1	No
1029	66	Male	30	AF	Rivaroxaban 20mg/24h	Atrium closure + valve repair	Low (2)	No	High	3	Yes (Therapeutic)	No restart	Yes (Therapeutic)
1063	83	Male	21	AF	Apixaban 2.5mg/12h	Pacemaker placement	High (7)	No	Low	2	No	1	No

Abbreviations: AF, atrial fibrillation; BMI, body mass index (kg/m^2); DOAC, direct oral anticoagulant; ERCP, endoscopic retrograde cholangiopancreatography; ID, patient identification; IS, ischemic stroke; LMWH, low molecular weight heparin, (therapeutic or prophylactic dose); TUR, transurethral resection; VTE, venous thromboembolism.
[a]See Supplementary Material S1.
[b]See ►**Supplementary Table S3**.
[c]During the 30-d follow-up.

Supplementary Table S7 Detailed characteristics of death patients

ID	Age (y)	Gender	BMI	Death etiology	DOAC indication	DOAC dose	Type of surgery	Thrombotic risk[a] (CHA_2DS_2-VASc score)	Type of thrombotic event	Surgery bleeding risk[b]	Bleeding event[a]	Preoperative DOAC withdrawal time (d)	Preoperative LMWH (dose)	Postoperative DOAC restart time (d)[c]	Postoperative LMWH (dose)
167	81	F	23	Sepsis	AF	Apixaban 5mg/12h	Total thyroidectomy	Moderate (5)	No	Moderate	No	3	No	No restart	Yes (Prophylactic)
188	78	M	28	Hearth failure	AF	Rivaroxaban 15mg/24h	Urethrotomy	Moderate (6)	No	Low	No	2	No	2	Yes (Prophylactic)
250	60	F	25	Cerebral tumor	AF	Dabigatran 110mg/12h	Craniotomy	Low (3)	No	High	No	13	Yes (Therapeutic)	No restart	No
434	81	F	22	Septic shock	AF	Apixaban 5mg/12h	Below-knee amputation	Moderate (5)	No	Moderate	No	2	Yes (Therapeutic)	No restart	No
441	76	M	27	Pneumonia	AF	Rivaroxaban 20mg/24h	Hepatic resection	Moderate (5)	No	Moderate	No	3	No	6	Yes (Prophylactic)
485	69	F	26	Hemorrhage	AF	Rivaroxaban 20mg/24h	Cholangio-carcinoma	Low (2)	No	High	Yes (Major)	2	Yes (Therapeutic)	No restart	No
504[d]	80	M	33	Hemorrhage	AF	Apixaban 2.5mg/12h	Septic hip arthritis	Moderate (5)	No	Low	No	2	No	No restart	Yes (Therapeutic)
513	76	F	40	Sepsis	AF	Apixaban 5mg/12 h	Inguinal adenopathy exeresis	Low (4)	No	Low	No	3	Yes (Prophylactic)	<24 h	No
525	80	M	28	Pneumonia	AF	Rivaroxaban 20mg/24h	Lobectomy	Low (3)	Yes (IS)	Moderate	No	2	No	No restart	Yes (Therapeutic)
547	82	M	34	Ischemic stroke	AF	Dabigatran 110mg/12h	Colonoscopy	Low (4)	Yes (IS)	Low	No	3	Yes (Therapeutic)	No restart	Yes (Therapeutic)
587	90	F	18	Hemorrhage	AF	Apixaban 5mg/12h	Hip fracture	High (7)	No	Moderate	Yes (Major)	6	Yes (Prophylactic)	No restart	Yes (Prophylactic)
722	85	M	26	Hearth failure	AF	Dabigatran 110mg/12h	Hip fracture	Moderate (6)	No	Moderate	Yes (Minor)	5	Yes (Prophylactic)	5	Yes (Prophylactic)
794	77	F	24	Hearth failure	AF	Apixaban 2.5mg/12h	Mastectomy	Moderate (5)	No	Moderate	No	3	Yes (Prophylactic)	No restart	Yes (Prophylactic)
1030	78	M	19	Septic shock	AF	Apixaban 2.5mg/12h	ERCP	High (7)	No	Moderate	No	3	Yes (Prophylactic)	No restart	Yes (Prophylactic)

Abbreviations: AF, atrial fibrillation; BMI, body mass index (kg/m^2); DOAC, direct oral anticoagulant; ERCP, endoscopic retrograde cholangiopancreatography; F, female; ID, patient identification; IS, ischemic stroke; LMWH, low molecular weight heparin, (therapeutic or prophylactic dose); M, male; VTE, venous thromboembolism.

[a]See Supplementary Material S1.

[b]See ►**Supplementary Table S3**.

[c]During the 30-d follow-up.

[d]This patient underwent a cardiac surgery 24 d after his primary surgery (septic hip arthritis). The etiology of the death was a severe postoperative hemorrhage after the cardiac procedure. The trial scientific committee considered this hemorrhage not a major hemorrhagic event related with the administration of the DOAC because it was not resumed after surgery and the patient was under an LMWH at therapeutic dose.
